# Low bacterial contamination rate in platelet components following primary culture screening: a Saudi Arabian experience

**DOI:** 10.3389/fmed.2026.1865609

**Published:** 2026-07-29

**Authors:** Mohammed Battah Alenazi, Muhammad Raihan Sajid, Salman Aldosari, Abdulwahab Binjomah, Jana H. Husein, Rasha M. Khayata, Liyan Afaneh, Badr Elwy

**Affiliations:** 1 College of Medicine, Alfaisal University, Riyadh, Saudi Arabia; 2 Riyadh Central Blood Bank, Blood Transfusion Service, Health Support Service Center, MOH, Riyadh, Saudi Arabia; 3 Department of Pathology, College of Medicine, Alfaisal University, Riyadh, Saudi Arabia; 4 Bacterial Diseases and Special Pathogens Department, Public Health Authority, Riyadh, Saudi Arabia

**Keywords:** BacT/ALERT VIRTUO, bacterial contamination, culture-confirmed, hemovigilance, platelets, positive predictive value (PPV), Saudi Arabia

## Abstract

**Background:**

Platelet storage at room temperature (20–24° C) with agitation facilitates bacterial proliferation. Despite global standards for culture-based screening, there is a lack of component-level contamination data within the Saudi Arabian context.

**Methods:**

We conducted a retrospective review of 28,416 post-donation platelet components at the Riyadh regional laboratory (2020–2021) to determine the culture-confirmed bacterial contamination rate. Screening for bacterial contamination utilized the BacT/ALERT^®^ VIRTUO system at 24 ± 2 h post-collection. The primary outcome was to measure the confirmed contamination rate per 1,000 components; secondary outcomes included the positive predictive value (PPV) of initial screening and organism distribution. Screen-positive units were quarantined and underwent a formal confirmation algorithm via repeat culture and subculture.

**Results:**

Out of 28,416 components, 49 (0.17%) were initially screen-positive. Twenty-five cases were culture-confirmed, yielding a contamination rate of 0.88 per 1,000 components (95% CI: 0.54–1.23) and a Positive Predictive Value (PPV) of 51.0%. Skin commensals predominated, led by *Staphylococcus epidermidis* (28.0%) and *Corynebacterium* spp. (16.0%).

**Conclusion:**

The confirmed contamination rate aligns with international benchmarks for primary culture programs. These findings suggest that implementing advanced strategies like Large-Volume Delayed Sampling (LVDS) or near-issue secondary testing could further reduce the residual risk posed by slow-growing skin flora.

## 1 Introduction

Platelet transfusions are indispensable in hematology–oncology, major surgery, trauma, and critical care. Unlike red blood cells, platelets are stored at room temperature with continuous agitation, a condition that favors bacterial replication, despite diversion pouches and skin antisepsis (1, 2). Consequently, bacterial contamination of platelet components remains a leading residual infectious risk of transfusion. Most confirmed cases originate from skin commensals introduced during collection, particularly *Staphylococcus epidermidis* (1, 3, 4). To reduce such risk, culture-based post-donation screening became standard practice in many countries and is recommended by professional bodies (2).

While these screening practices are well-established globally, regional data remains limited. Saudi publications have focused mainly on broader hemovigilance indicators—such as transfusion reaction and wastage rates—rather than component-level, culture-confirmed contamination in platelets (5, 6). A recent single-center observational study from Saudi Arabia reported a bacterial contamination rate of 0.28% among platelet concentrate units, further supporting the need for local data aggregation (6).

This evidence gap limits benchmarking and targeted quality improvement, particularly regarding the positive predictive value (PPV) of the initial screen, organism distribution, and contamination trends by component type. Generating local, component-level estimates is essential to directly inform local policy makers by revising operational policy and imparting staff education and training. The findings from this study are expected to directly inform local transfusion policy by establishing a national evidence-based benchmark for bacterial contamination rates, guiding decisions regarding the implementation of pathogen reduction technologies or large-volume delayed sampling strategies, and strengthening hemovigilance education for collection staff on skin disinfection protocols.

This retrospective study was conducted at the Regional laboratory and Blood bank in Riyadh covering the 2020–2021 period to address this specific local evidence gap. Our main goal was to estimate the rate of culture-confirmed bacterial contamination per 1,000 post-donation platelet components processed within the designated timeframe, quantify the positive predictive value (PPV) of the initial culture screen, and describe the organism profile among culture-confirmed positives and assess differences by component type.

## 2 Materials and methods

### 2.1 Study design

This study adopted a retrospective laboratory quality review at the Regional Laboratory and Blood Bank, Riyadh, Saudi Arabia, covering January 2020–December 2021. The service follows American Association of Blood Banks (AABB)-aligned standard operating procedures (SOPs) for platelet screening, quarantine, discard, and traceability (7, 8). The service also uses an integrated Laboratory information system (LIS) to capture component production and microbiology data (9). These facilities are equipped with the necessary technology and infrastructure to perform comprehensive platelet assessments and maintain detailed records of platelet donations and testing. Ethical approval from the Institutional Review Board (IRB) of the King Saud Medical city, Ministry of Health, Kingdom of Saudi Arabia (Ref: H1RE-10-Oct22-01) was received on 10th October,2022. Due to the retrospective nature of this laboratory quality review using anonymized data, the Institutional Review Board granted a waiver of informed consent from individual donors. All donor and patient data were anonymized to ensure confidentiality.

### 2.2 Sample selection

The study included all post-donation platelet components prepared for clinical issues during the study period. These comprised single-donor aphaeresis platelets (SDP) and random-donor platelets (RDP) derived from whole blood via buffy-coat or Platelet rich plasma (PRP) methods. We excluded components with invalid culture records, non-microbiological discards—such as label damage or temperature excursions—and units returned after expiration. Red blood cells and plasma were also omitted to prevent denominator drift and ensure platelet-specific inference.

Sample size justification: No formal sample size calculation was performed as this was a complete enumeration of all eligible platelet components processed during the 24-month study period. With 28,416 components screened, the precision around the contamination rate estimate (95% CI width of 0.69 per 1,000) is adequate for benchmarking purposes. During the study period, aphaeresis platelets (SDP) comprised 10,842 components (38.2%) and random-donor platelets (RDP) comprised 17,574 components (61.8%), reflecting the center’s product mix.

### 2.3 Laboratory screening and confirmation protocols

Prior to collection, donor identification and arm-preparation checklists were documented. Blood samples were then collected via venipuncture at the antecubital fossa following a strict alcohol–chlorhexidine antisepsis protocol ( ≥ 30 s contact time). To further reduce contamination risk, a diversion pouch was utilized before filling the main bag. Collected platelets were prepared as RDP or SDP according to SOPs, with all device data recorded in the LIS. Finally, components were stored at 20–24 °C with continuous mechanical agitation and routine temperature monitoring.

At 24 ± 2 h post-collection, primary bacterial screening was performed using the BacT/ALERT VIRTUO system (bioMérieux, France) utilizing both aerobic and anaerobic bottles – specifically BacT/ALERT PF aerobic (FA) and anaerobic (FN) bottles. Inoculation volumes strictly adhered to standard protocols: a minimum of 10 mL total for random-donor platelets (divided equally between bottles) and a minimum of 3 mL per bottle for single-donor platelets. As Per policy, a retention sample consisting of a 10 cm segment of plasma from each platelet unit was kept at room temperature for 7 days to enable repeat culture if the initial screen flagged positive. The use of different inoculation volumes for RDP (10 mL total) versus SDP (6 mL total) represents a potential limitation in detection sensitivity. Lower volume cultures (3 mL per bottle for SDP) have been shown to reduce the probability of capturing low-inoculum contamination compared to the 8 mL per bottle recommended by some guidelines. However, our protocol adhered to manufacturer specifications for the BacT/ALERT system and local SOPs consistent with AABB standards. Any volume-related reduction in sensitivity would bias toward underestimation of true contamination, meaning our reported rate of 0.88/1,000 should be considered a conservative estimate. Bottles were incubated for 5–7 days under automated growth monitoring. During the study period, primary culture constituted the sole bacterial risk-control strategy, as neither pathogen reduction technology nor large-volume delayed sampling was in routine use.

Any initial automated growth flag triggered immediate component quarantine. To define a “culture-confirmed positive,” secondary testing was mandated via: (1) repeat culture from a retained tubing segment (the integral segments attached to the platelet bag, which are representative of bag contents but limited by small volume [1–2 mL] and potential bacterial adhesion to tubing surfaces); and (2) subculture on solid media (blood, chocolate, or anaerobic agar) using the blood-broth mix from the initially flagged BacT/ALERT bottle. Subculture from the flagged bottle has high sensitivity because it samples broth containing actively growing bacteria. Final organism identification was achieved using MALDI-TOF mass spectrometry and standard biochemical assays. A component was strictly classified as a culture-confirmed positive only when growth was demonstrated on repeat testing with concordant organism identification. When the initial screen flagged positive, repeat testing was performed using both methods in parallel. Confirmation required growth in at least one of these secondary tests with organism identification matching the initial flag.

### 2.4 Defined outcomes and statistical analysis

The defined primary outcome was the confirmed contamination rate per 1,000 platelet components; the secondary outcome was the PPV of the initial screen in predicting a confirmed positive; the tertiary outcome was organism distribution among confirmed positive components and comparison of contamination rates between RDPs and SDPs. Analyses were performed using IBM SPSS v29.0. Contamination rates per 1,000 components were calculated with exact binomial 95% confidence intervals using the Clopper-Pearson method. The positive predictive value (PPV) was calculated as (true positives/[true positives + false positives]) × 100, with exact 95% CIs. For comparison of contamination rates between RDP and SDP components, we calculated the rate ratio (RR) with 95% confidence intervals using Poisson regression. Monthly contamination rates were examined using a control chart (p-chart) with 3-sigma control limits to identify any temporal trends or special cause variation, though no significant trends were observed.

## 3 Results

### 3.1 Study throughput and contamination rates

During the study period, a total of 28,416 post-donation platelet components underwent primary culture screening. All 28,416 components completed the full 5–7-day incubation protocol under continuous automated monitoring as described. The initial primary screen yielded 49 positive flags, representing a rate of 1.72 per 1,000 components (0.17%). All 49 screen-positive components were placed under mandatory quarantine per standard operating procedures, ensuring no contaminated units were issued for patient use. Contamination was confirmed through culture of the quarantined components, which yielded 25 true positives from the 49 initial flags. Related co-components, such as red blood cells and plasma, that were derived from the contaminated donations were traced and removed from inventory in accordance with corrective and preventive action (CAPA) protocols. Of these co-components (total 50 units: 25 RBC and 25 plasma), none demonstrated visual evidence of hemolysis or discoloration. Confirmatory culture of quarantined co-components was not routinely performed per protocol, as bacterial contamination in platelet components does not reliably predict contamination of simultaneously collected RBC or plasma units due to different storage conditions (refrigeration for RBC inhibits bacterial growth). This resulted in a final culture-confirmed contamination rate of 0.88 per 1,000 components (95% CI: 0.54–1.23). Contamination rates can be seen in Table 1.

**TABLE 1:** Screening results of the platelets culture.

Metric	Count	% of Total	per 1,000 (95% CI)
**Total components screened**	28,416	–	–
**Initial screen positives**	49	0.17%	1.72
**Culture-confirmed positives**	25	0.09%	0.88 (0.54–1.23)
**PPV of initial screen**	–	–	51.0% (≈37–65%)

### 3.2 Positive predictive value (PPV) and incubation dynamics

The positive predictive value (PPV) of the initial automated culture screen was 51.0% (95% CI: 37–65%). This reflects a known balance between early false-positives, instrument signals without repeat growth, and true positives meeting confirmation criteria. Notably, a subset of culture flags occurred after day five of incubation. Under the 24-hour primary sampling algorithm, these delayed positives reflect the occurrence of true bacterial proliferation during storage from low-level contamination or early false negatives at 24 h due to low inoculum.

### 3.3 Organism profile

Coagulase-negative staphylococci (CoNS) predominated, accounting for 17 of 25 isolates (68.0%), including *Staphylococcus epidermidis* (28.0%), Staphylococcus capitis (12.0%), Staphylococcus hominis (8.0%), Staphylococcus warneri (8.0%), and single isolates of Staphylococcus haemolyticus and Staphylococcus gallinarum (4.0% each). Corynebacterium spp. (12.0%) were the next most common. The remaining 28.0% of cases comprised seven distinct single isolates; the complete distribution of all identified organisms is detailed in Table 2.

**TABLE 2:** Distribution of identified organisms in platelet cultures.

Results	Counts	% of Total	Cumulative %
*Staphylococcus epidermidis*	7	28.0%	28.0%
*Staphylococcus capitis*	3	12.0%	40.0%
*Corynebacterium* spp.	3	12.0%	52.0%
*Staphylococcus hominis*	2	8.0%	60.0%
*Staphylococcus warneri*	2	8.0%	68.0%
*Staphylococcus haemolyticus*	1	4.0%	72.0%
*Staphylococcus gallinarum*	1	4.0%	76.0%
Coagulase-negative *Staphylococcus* (unspeciated)	1	4.0%	80.0%
*Bacillus* spp.	1	4.0%	84.0%
*Serratia marcescens*	1	4.0%	88.0%
*Streptococcus parasanguinis*	1	4.0%	92.0%
*Streptococcus viridans*	1	4.0%	96.0%
*Streptococcus* spp. (unspeciated)	1	4.0%	100.0%
Total	25	100%	

### 3.4 Contamination rates by component type

Among the 28,416 components screened, 10,842 (38.2%) were SDP and 17,574 (61.8%) were RDP. The culture-confirmed contamination rate was 0.55 per 1,000 SDP components (6/10,842; 95% CI: 0.20–1.20) compared with 1.08 per 1,000 RDP components (19/17,574; 95% CI: 0.65–1.69). The rate ratio for RDP versus SDP was 1.96 (95% CI: 0.78–5.47), suggesting an approximately two-fold higher contamination rate among RDP components; nevertheless, this difference was not statistically significant (*p* = 0.15). Although the observed trend may be related to differences in collection and processing procedures between component types, the study was not designed to identify the underlying causes of this association.

### 3.5 Analysis of non-confirmed screen-positive bottles

Of the 49 initially screen positive flagged bottles, 24 (49.0%) were not confirmed as true contamination results. All 24 underwent repeat testing, comprising culture of retained tubing segments and subculture of broth from the flagged bottles onto solid media. In every case, both confirmatory procedures produced negative results for bacterial growth. No discrepant findings (e.g., recovery of a different organism on repeat testing) were detected.

Consequently, these 24 cases were classified as false positive screening signals, presumably attributable to instrument related artifacts, transient detection signals, or very low level bacterial contamination that was not reproducible on repeat testing.

All 25 confirmed-positive components yielded bacterial growth on subculture of the flagged bottle broth onto solid media. The recovered isolates were then identified using MALDI-TOF mass spectrometry and standard biochemical assays. Repeat culture from retained tubing segments was also performed for all 25 components; however, this method did not yield growth in any case. This finding is likely attributable to the small volume of the tubing segments (1–2 mL) which may reduce the sensitivity of this method compared to subculture of the actively growing broth culture.

### 3.6 Time-to-positivity analysis

Among the 25 confirmed-positive components, the median time-to-positivity (TTP) was 38.2 h (range: 18.5–162.0 h). Eleven confirmed positives (44.0%) flagged positive after 72 h of incubation, including 4 (16.0%) after day 5 (range 120–162 h). All false-positive flags (*n* = 24) occurred within the first 48 h of incubation (median TTP 22.4 h), suggesting that late flags ( > 72 h) are more likely to represent true contamination with slow-growing organisms. These data support the value of extended incubation protocols (5–7 days) rather than early termination at 3–4 days.

## 4 Discussion

### 4.1 Principal findings and global context

The confirmed contamination rate of 0.88/1,000 observed in this large-scale screening of 28,416 platelet components aligns perfectly with international standards for centers using 24-hour primary culture programs (2, 10, 11). While global blood transfusion safety measures in recent literature frequently report persistent residual risks despite advances in donor arm antisepsis and diversion pouches (2, 12, 13), equivalent large-scale data from within the Kingdom of Saudi Arabia remains relatively underrepresented. This outcome not only confirms that local screening protocols are performing effectively at an international standard to minimize transfusion-transmitted infections, but it also establishes a robust regional benchmark for platelet safety and quality control.

### 4.2 Positive predictive value and incubation limitations

The observed PPV of 51% indicates that nearly half of the initial culture positive signals could not be confirmed on repeat testing. These non-confirmed signals may have resulted from instrument related artifacts, transient detection signals, technical variability during laboratory processing (technical error), or bacterial concentrations below the threshold of reproducible detection (4). As the exact cause of each non-confirmed signal could not be established, these explanations should be considered possible rather than conclusive.

The high proportion of non-confirmed screening signals revealed in this study (49% of initially positive instrument flags) is consistent with previous studies. Corean et al. (14) demonstrated that false-positive signals in platelet bacterial screening programs are common, reflecting the balance between maximizing sensitivity and minimizing the risk of missing contaminated components. In our study, among 28,416 components screened, we observed 25 true-positive and 24 false-positive results. When calculated using the same denominator (28,416), the true-positive rate was 0.088% (25/28,416) and the false-positive rate was 0.084% (24/28,416), yielding a false-positive to true-positive ratio of 0.96 (24/25). This near-equivalence of false-positive and true-positive rates highlights the importance of confirmatory testing before component disposition, as relying solely on the initial instrument alert resulted in the unnecessary discard of 24 sterile platelet units (10, 14).

Furthermore, sampling at 24 h carries an inherent risk of false negative results, particularly when the initial bacterial load is very low or when contaminating organisms grow slowly (15). Under these conditions, bacterial replication may not reach detectable levels during the initial culture period, delaying detection until later in storage (2). This highlights an important biological limitation of relying solely on early primary culture for platelet bacterial screening. The relatively high proportion of non-confirmed positive signals observed in this study also emphasizes the importance of a robust confirmatory bacterial testing strategy before final decisions regarding component disposition are made (16).

### 4.3 Microbiological profile and contamination dynamics

The microbial isolates were heavily dominated by skin commensals, mainly S. epidermidis (28%) and Corynebacterium spp. (16%), thus confirming the donor’s skin as the primary contamination source (6). Despite the continuous improvement of blood supply safety through rigorous donor screening and hemovigilance, bacterial contamination of platelets remains a critical and persistent challenge for transfusion-transmitted infections (TTIs) (17). Extensive multi-year surveillance supports this ongoing risk, with large-volume delayed sampling (LVDS) and universal screening protocols revealing confirmed platelet positivity rates of 0.18%, frequently involving slow-growing flora like Cutibacterium acnes (18). The persistence of these contaminants is further enhanced by their ability to adapt to the physiological stressors of the platelet storage environment (19). Pathogens such as Staphylococcus aureus undergo significant genotypic and phenotypic modulation which leads to them entering a quiescent metabolic state, altering nutrient acquisition, and upregulating genes for cell wall modification and biofilm formation (19). These sophisticated adaptation strategies allow bacteria to resist immune clearance, survive pathogen reduction treatments, and escape detection during routine culture screening, posing a severe ongoing threat to transfusion recipients (19). Once introduced, the 20–24 °C storage environment with continuous agitation allows these organisms to proliferate to detectable levels (1). Conversely, rare isolates like Serratia marcescens suggest sporadic environmental or equipment-related sources, highlighting the need for continuous site audits alongside standard skin preparation (20). Current storage environments actively promote biofilm formation on platelet aggregates and on platelet bags under platelet storage conditions; specifically allowing for *Staphylococcus epidermidis* growth (3). Furthermore, retrospective investigations of suspected transfusion reactions confirm that other low-virulence skin flora, specifically Corynebacterium species and additional coagulase-negative staphylococci, are frequently isolated contaminants. This predominance reinforces that trace donor skin flora is the primary source of bacterial inoculation, utilizing these specific storage environments to persist within the blood components (6).

### 4.4 Contamination reduction techniques

To further mitigate this residual risk, blood centers can implement proactive Pathogen Reduction Technologies (PRT) that target the nucleic acids of infectious agents while preserving component efficacy. Current prominent systems utilize varied mechanisms: the INTERCEPT system (Cerus Corporation, Concord, CA, USA) employs amotosalen (S-59) and UVA light to form replication-blocking covalent cross-links, followed by the extraction of residual amotosalen via a Compound Adsorption Device (CAD). Alternatively, the Mirasol system (Terumo BCT, Lakewood, CO, USA) utilizes riboflavin (vitamin B2) and UV light to induce irreversible guanine oxidation; while this requires no extraction step, the generated reactive oxygen species (ROS) can impact sensitive proteins such as fibrinogen. Finally, the THERAFLEX UV system (Macopharma, Tourcoing, France) relies on direct UVC light exposure to induce pyrimidine dimers, avoiding the need for toxicological clearance but requiring specialized bags and high-intensity agitation for adequate light penetration (21–24). Alongside these primary systems, alternative methods utilizing methylene blue and Inactine (PEN110) provide additional avenues for pathogen inactivation (23).

### 4.5 Operational implications, strengths, and limitations

A key strength of this study is the large sample size (28,416 components) reflecting a real-world workflow that successfully prevented the transfusion of any contaminated units. However, limitations include the single-center design and the lack of molecular typing or differentiation between component types. An additional limitation is the suboptimal culture inoculation volume (10 mL total for RDP, 6 mL for SDP) compared to the 16 mL (8+8) recommended by some international guidelines. Lower volume reduces the probability of capturing very low-level contamination (< 1 CFU/mL), potentially leading to false-negative primary screens and underestimation of the true contamination rate. To further reduce the residual risk of slow-growing bacteria missed by 24-hour cultures, our facility should consider adopting Large-Volume Delayed Sampling (LVDS) or secondary testing closer to the time of issue (15, 19, 24).

Potential Impact of the COVID-19 Pandemic: This study period during 2020–2021, coincided with the COVID-19 pandemic, a period that carried significant changes to healthcare systems nationally and internationally, blood collection services, and laboratory operations worldwide. On one hand, enhanced infection prevention measures, such as stricter hand hygiene and environmental cleaning practices, may have helped reduce the risk of bacterial contamination. On the other hand, staffing shortages, increased workload demands, operational challenges, and changes in blood collection and inventory management may have affected contamination detection and reporting.

The overall impact of these factors cannot be determined from the available data. Nonetheless, the contamination rate observed in this study (0.88 per 1,000 platelet components) was comparable to rates reported before the pandemic, suggesting that any COVID-19 related effect on the findings was likely modest. However, these results should be interpreted within the context of the pandemic period, and future studies using post pandemic data would be valuable to confirm whether the findings remain consistent under routine operating conditions.

## 5 Conclusion

In conclusion, our retrospective review confirmed that post-donation platelet bacterial contamination was rare (0.88/1,000). Contamination was predominantly associated with coagulase-negative staphylococci (CoNS) and Corynebacterium species, consistent with skin-source inoculum. The PPV of ≈51% indicates that half of initial instrument flags are false positives (no growth on confirmatory testing). This finding supports continued use of a confirmation algorithm and transparent reporting of confirmed rates rather than raw instrument alerts, as discarding components based solely on initial flags would have resulted in unnecessary loss of 24 sterile units. To reduce platelet transfusion contamination, adopting pathogen reduction technologies can offer a proactive approach. These chemical and light-based mechanisms provide an essential layer of safety to ensure the sterility of transfusable units.

## Data Availability

The original contributions presented in this study are included in this article/supplementary material, further inquiries can be directed to the corresponding author.
